# Data on keratin expression in human cells cultured with Australian native plant extracts

**DOI:** 10.1016/j.dib.2016.02.069

**Published:** 2016-03-18

**Authors:** Damian H. Adams, Qingyao Shou, Hans Wohlmuth, Allison J. Cowin

**Affiliations:** a Regenerative Medicine, Future Industries Institute, University of South Australia, Mawson Lakes, South Australia 5095, Australia; b Southern Cross Plant Science, Southern Cross University, Lismore, New South Wales 2480, Australia; c Integria Healthcare, Gallans Road, Ballina, New South Wales 2478, Australia

## Abstract

Australian native plants have a long history of therapeutic use in indigenous cultures particularly for the treatment of wounds. We analysed 14 plant derived compounds from the species *Pilidiostigma glabrum*, *Myoporum montanum*, *Geijera parviflora*, and *Rhodomyrtus psidioides* for keratin 1, 5, 10 and 14 supporting the research article “Native Australian plant extracts differentially induce Collagen I and Collagen III in vitro and could be important targets for the development of new wound healing therapies” [5]. An in situ immunofluorescence assay was used in a 96 well tissue culture plate format to measure keratin expression in immortalised human keratinocytes (HaCaTs) exposed Australian native plant compounds to NMR spectra for the plant extracts are included in this article as is quantitative fluorescent intensity data of keratin 1, 5, 10 and 14 expression.

## **Specifications Table**

**Table:** 

Subject area	*Flora Chemistry, Biology*
More specific subject area	*Cell culture, microscopy, immunofluorescence*
Type of data	*Figures*
How data was acquired	*Fluorescence Microscopy, Olympus IX83 fluorescence microscope, Olympus DP80 camera, cellSens Dimension software*
Data format	*Analyzed*
Experimental factors	*Lyophilized plant extracts were dissolved in neat DMSO.*
Experimental features	*Plant extracts were obtained from native Australian plants. Human keratinocyte cell cultures (HaCaTs) were exposed to a DMSO concentration of 0.5% v/v, and 20 µM test plant compound for 60hrs in all tests. Cells were analyzed for the expression of Keratins 1, 5, 10, and 14 using insitu-immunofluorescence and measurements of fluorescence intensity of captured images.*
Data source location	*Mawson Lakes, Adelaide, South Australia, Australia.*
Data accessibility	*Data is with this article.*

## **Value of the data**

• We have developed a novel 96 well format in situ immunofluorescence assay for measuring the level of different proteins present in cells.
• This novel assay was used to determine the effect of Australian native plant extracts on these different keratins in keratinocytes.
• This assay could be a useful tool for the quantification of specific protein levels in large numbers of samples.

## 1 Data

Dataset provided in this article shows the NMR spectra for compounds extracted from native Australian plants species *Pilidiostigma glabrum*, *Myoporum montanum*, *Geijera parviflora*, and *Rhodomyrtus psidioides*. In addition to collagen synthesis [5], additional analysis was performed to determine keratin expression. Data is included that shows keratin 1, 5, 10 and 14 protein expression in the cultured human keratinocyte cell line (HaCaT).

## 2 Experimental design, materials and methods

### 2.1 Plant material

**Fig. 1:**
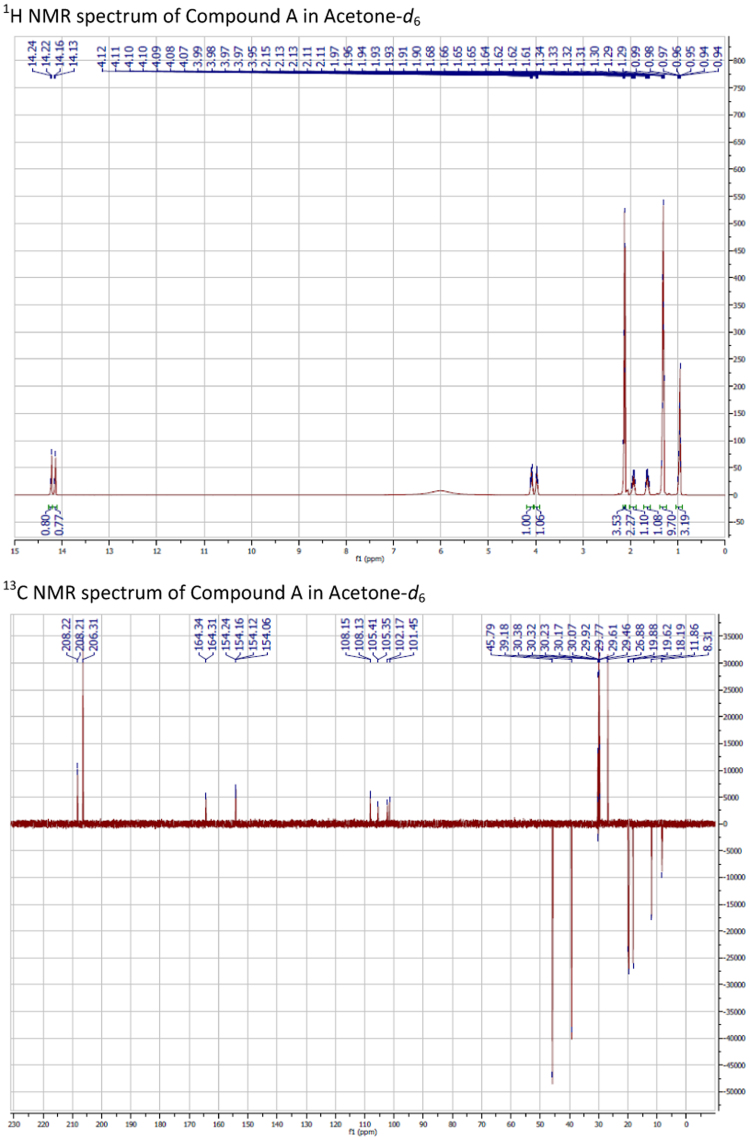

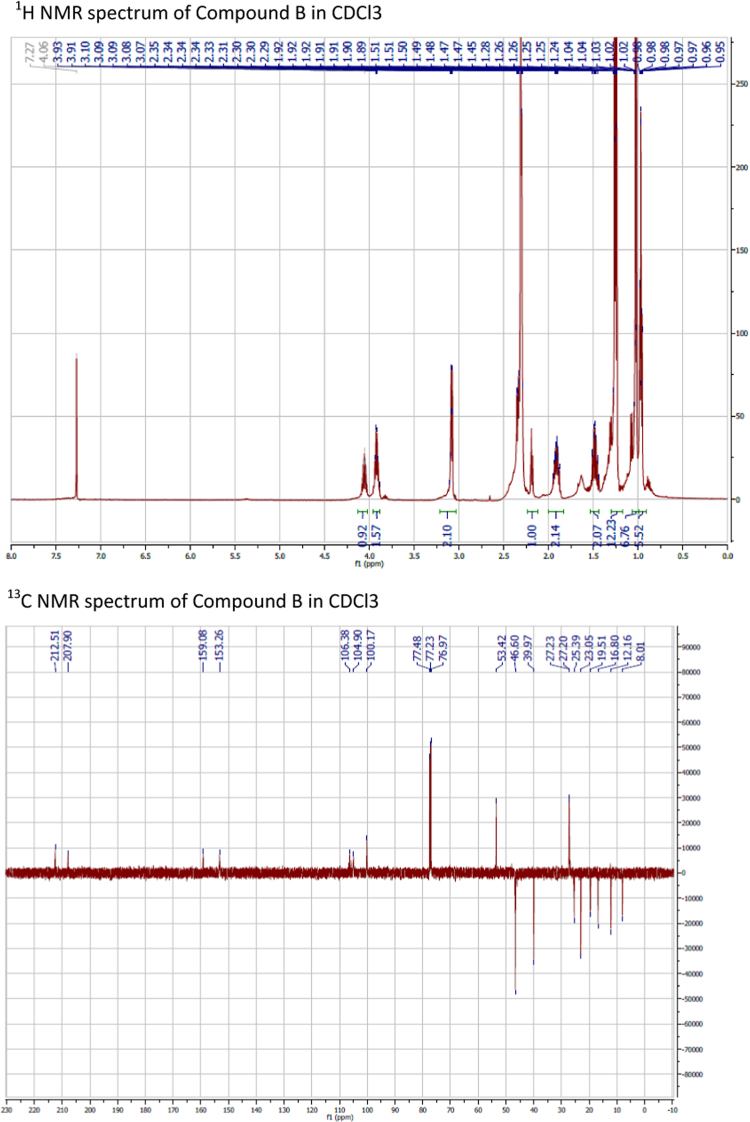

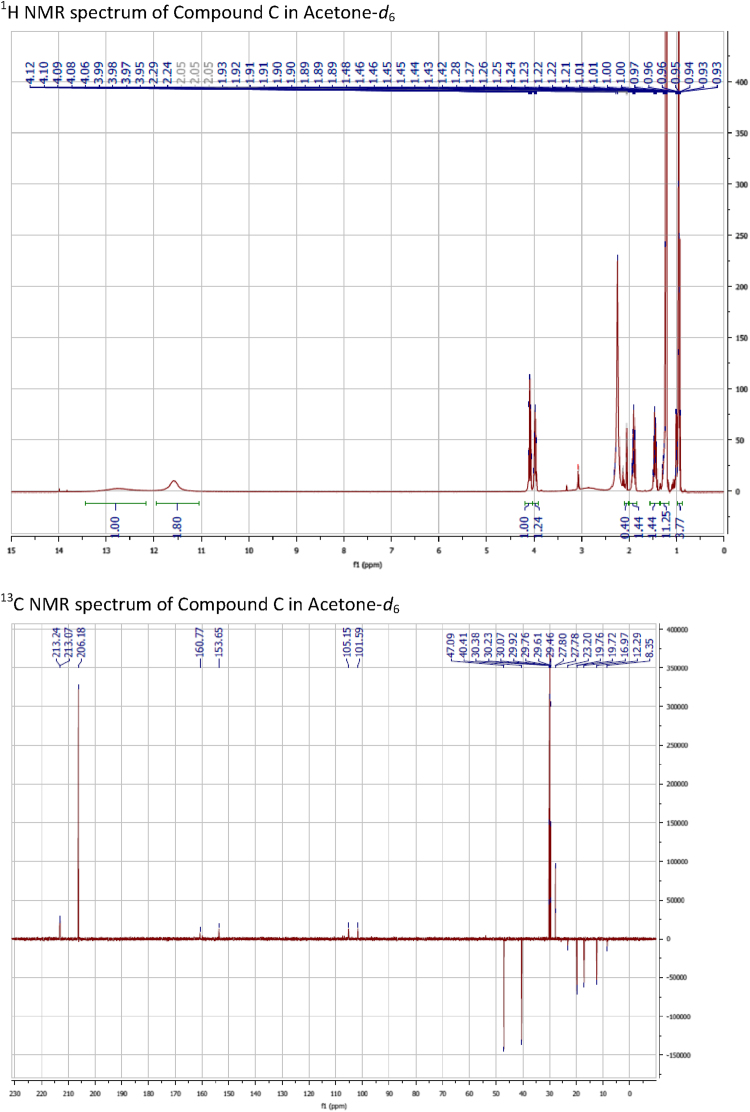

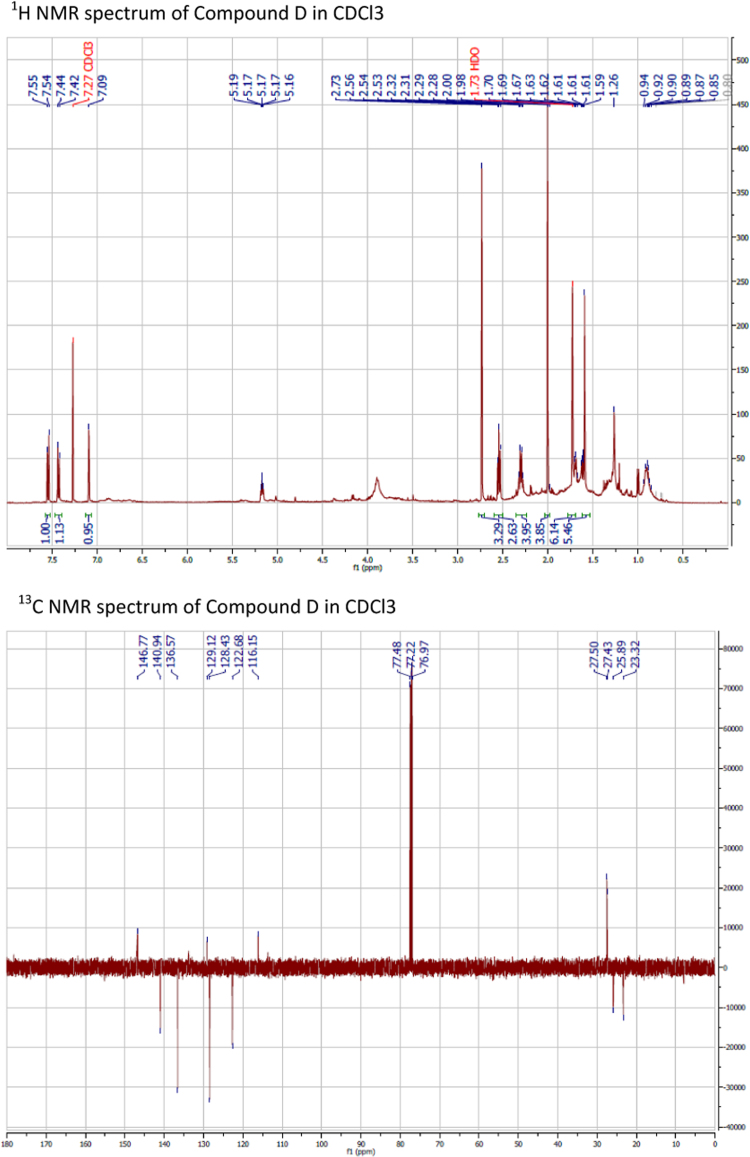

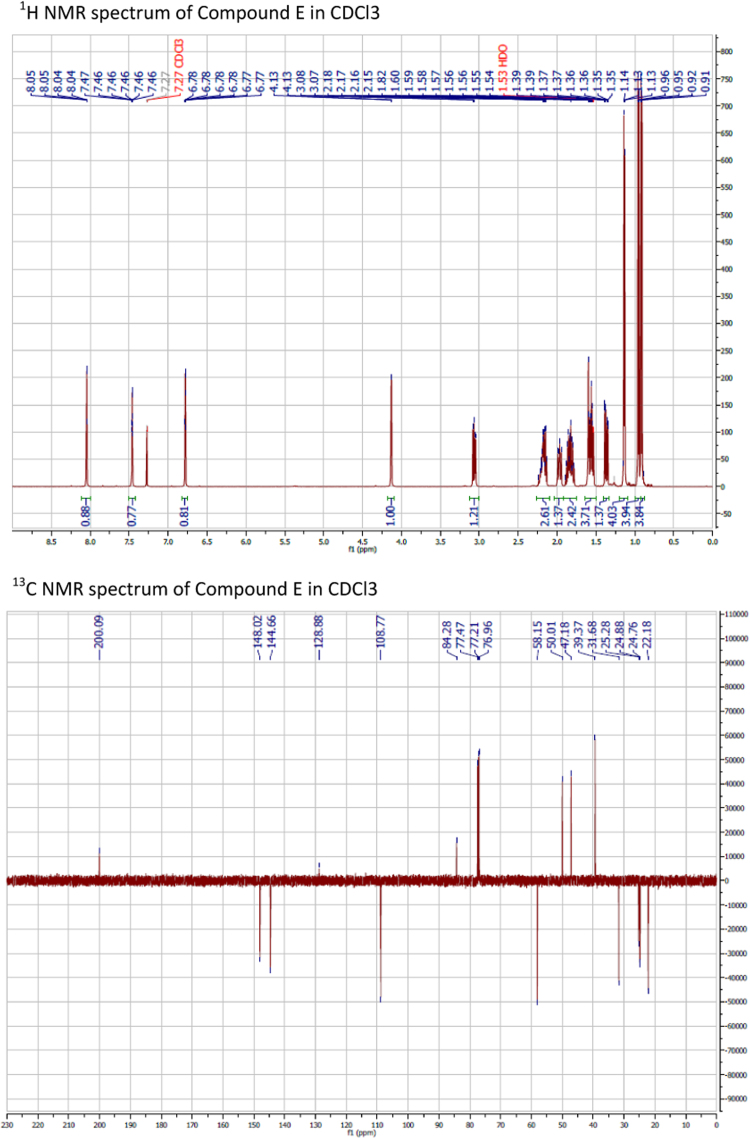

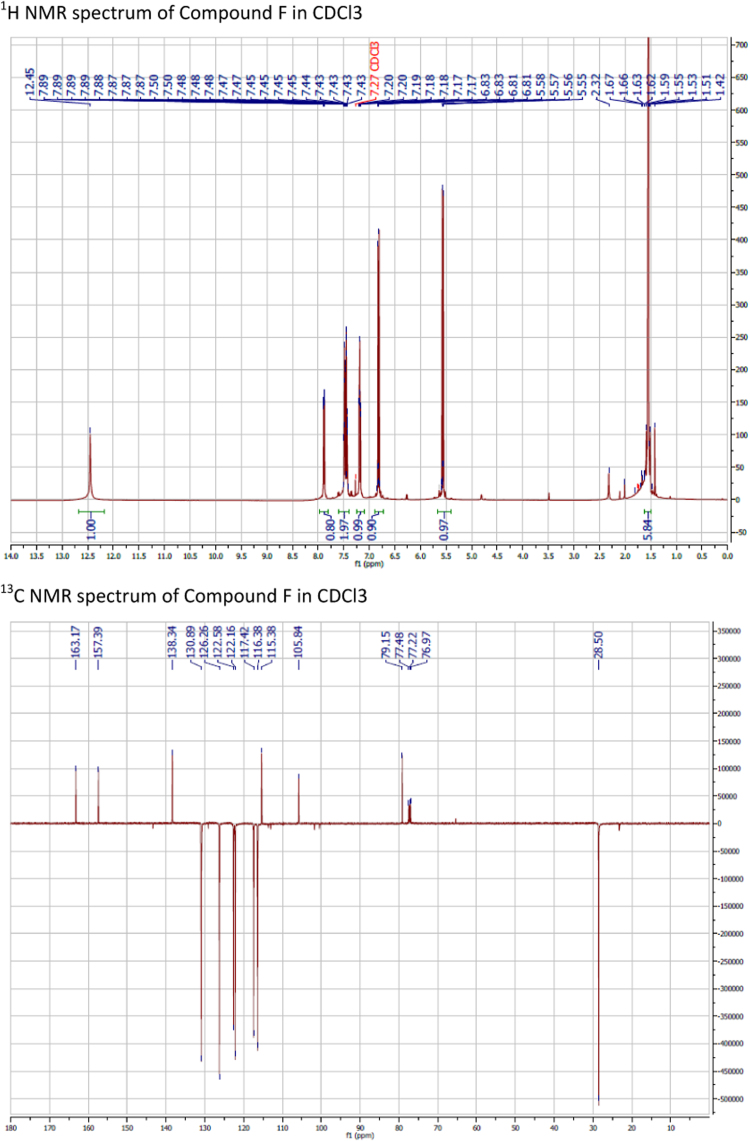

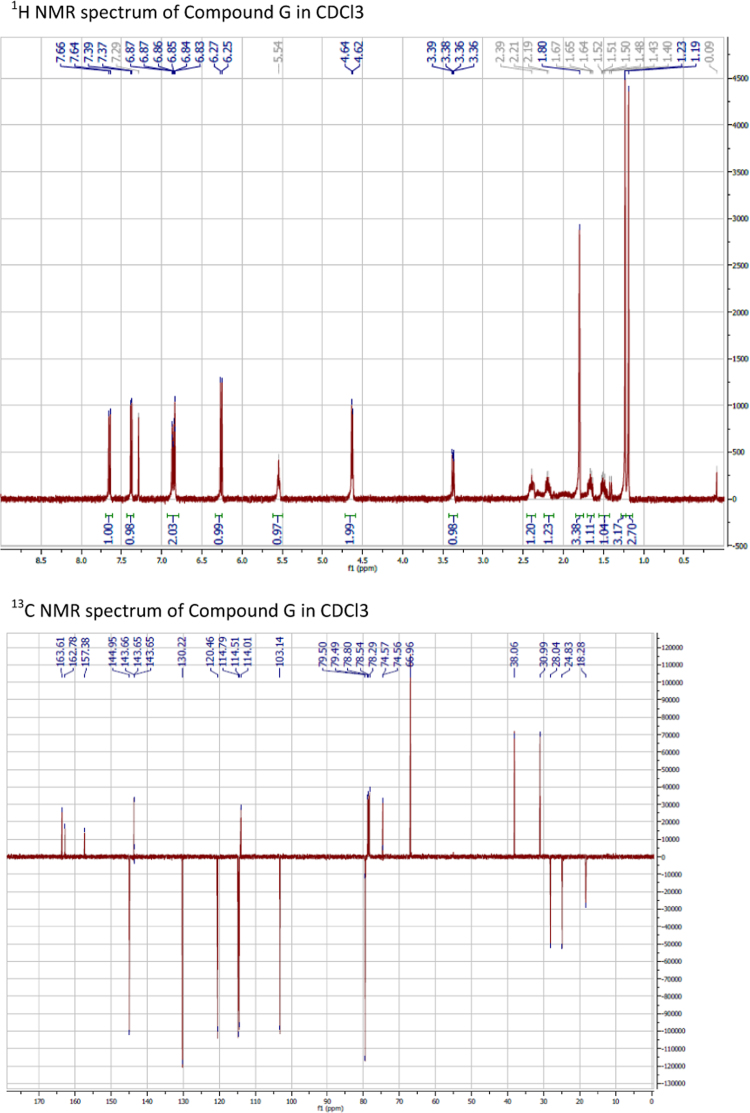

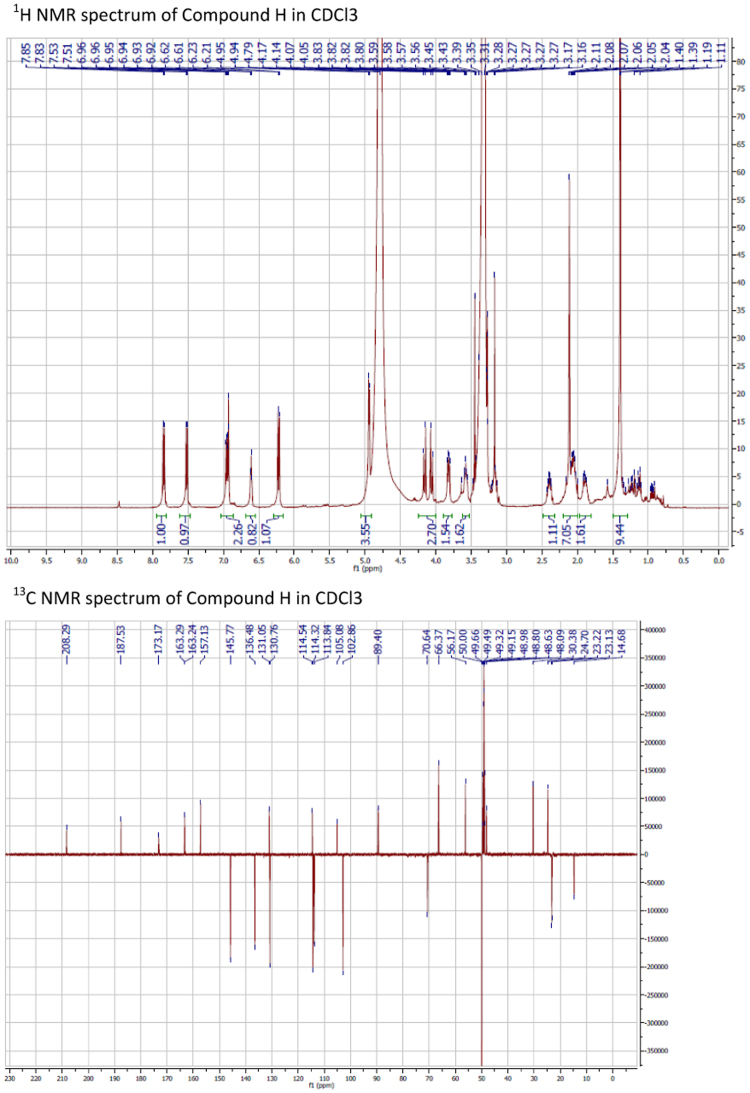

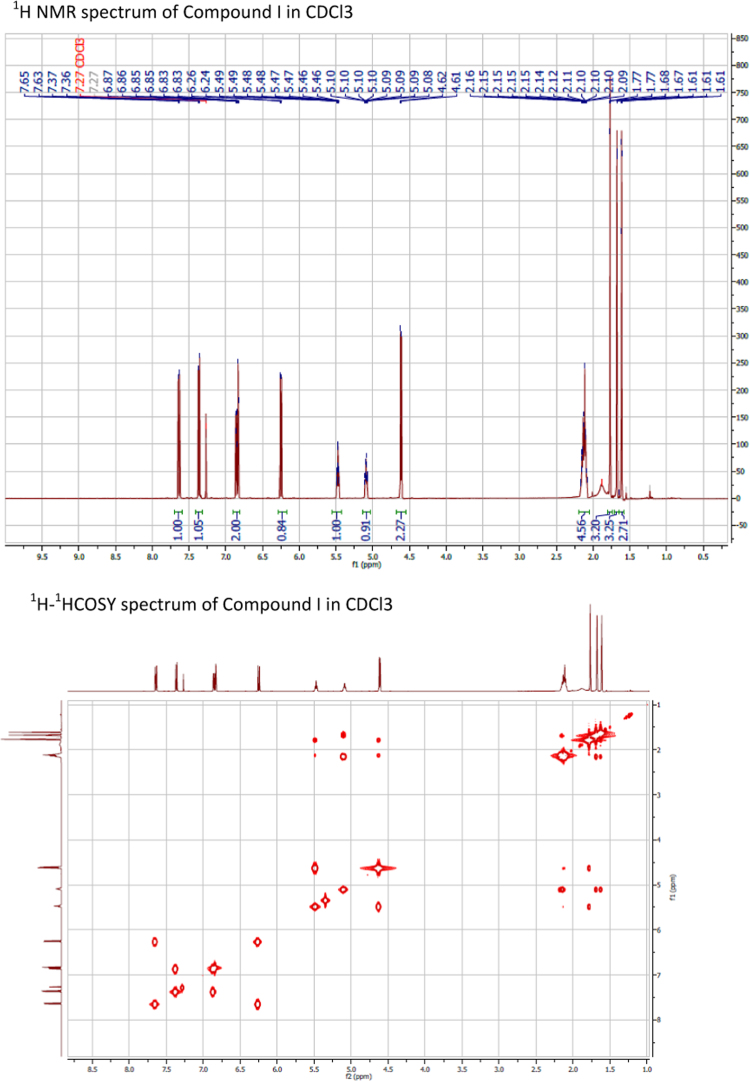

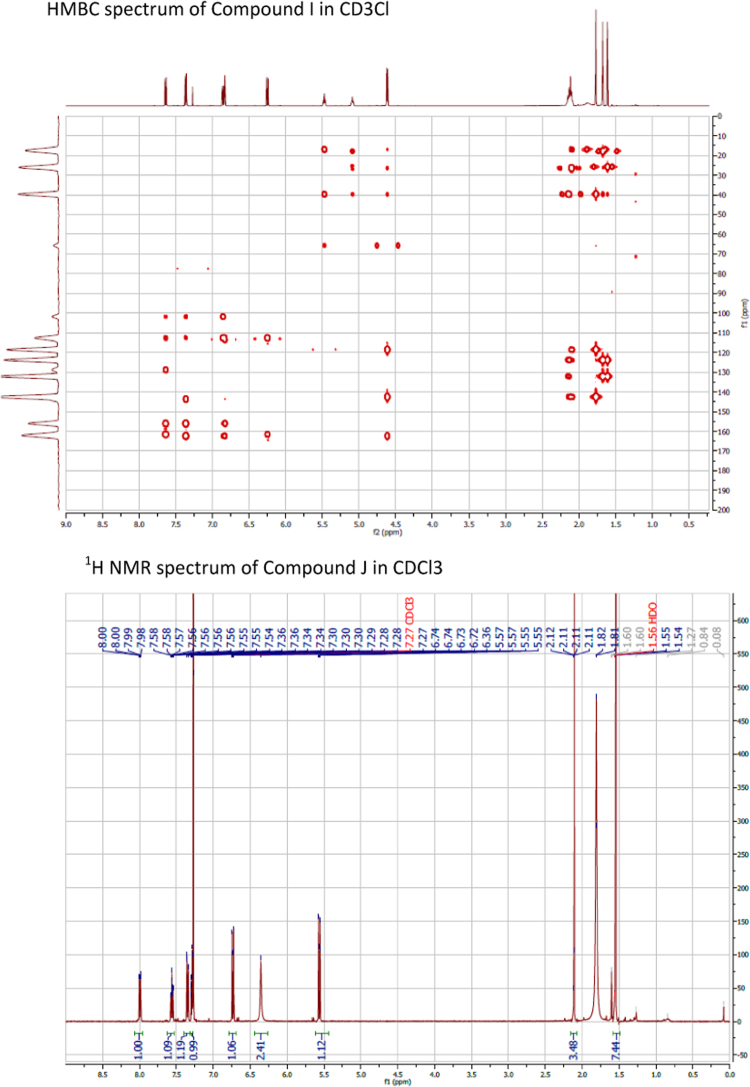

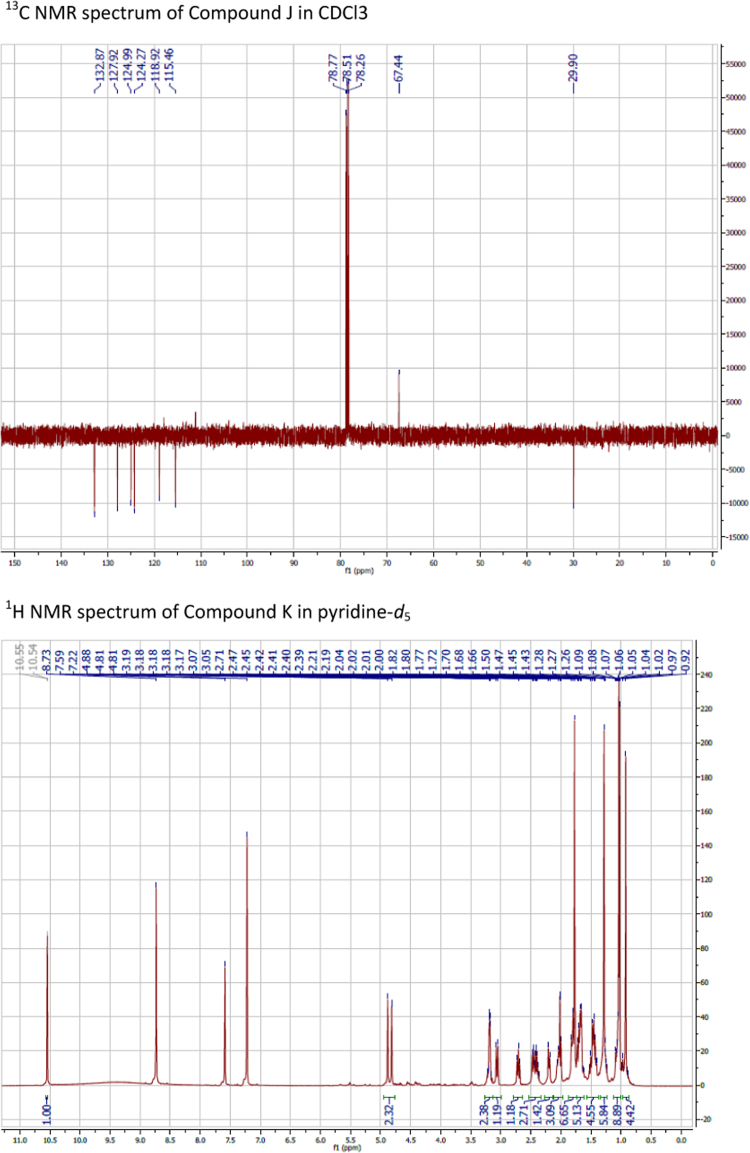

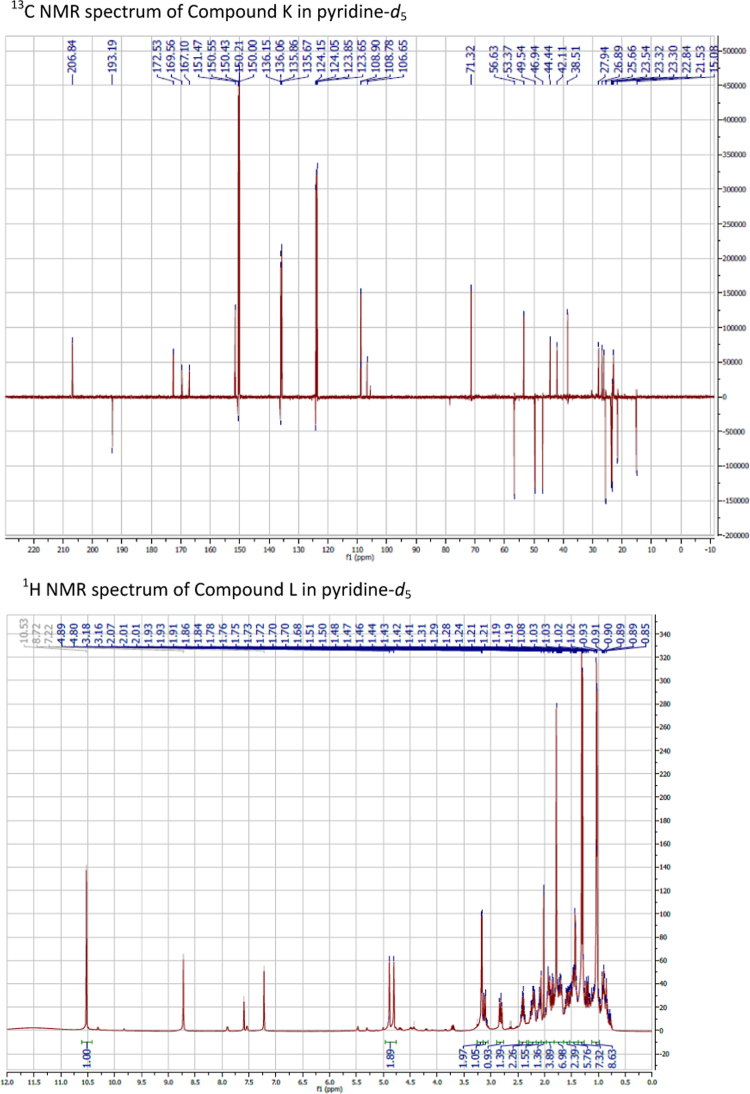

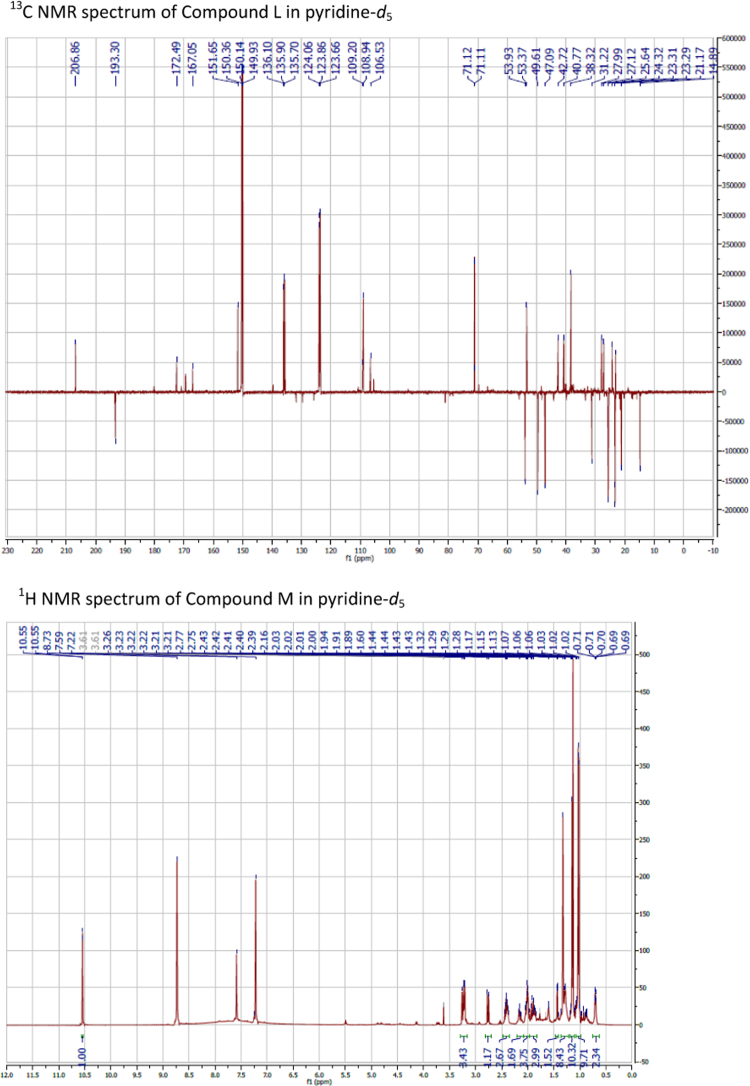

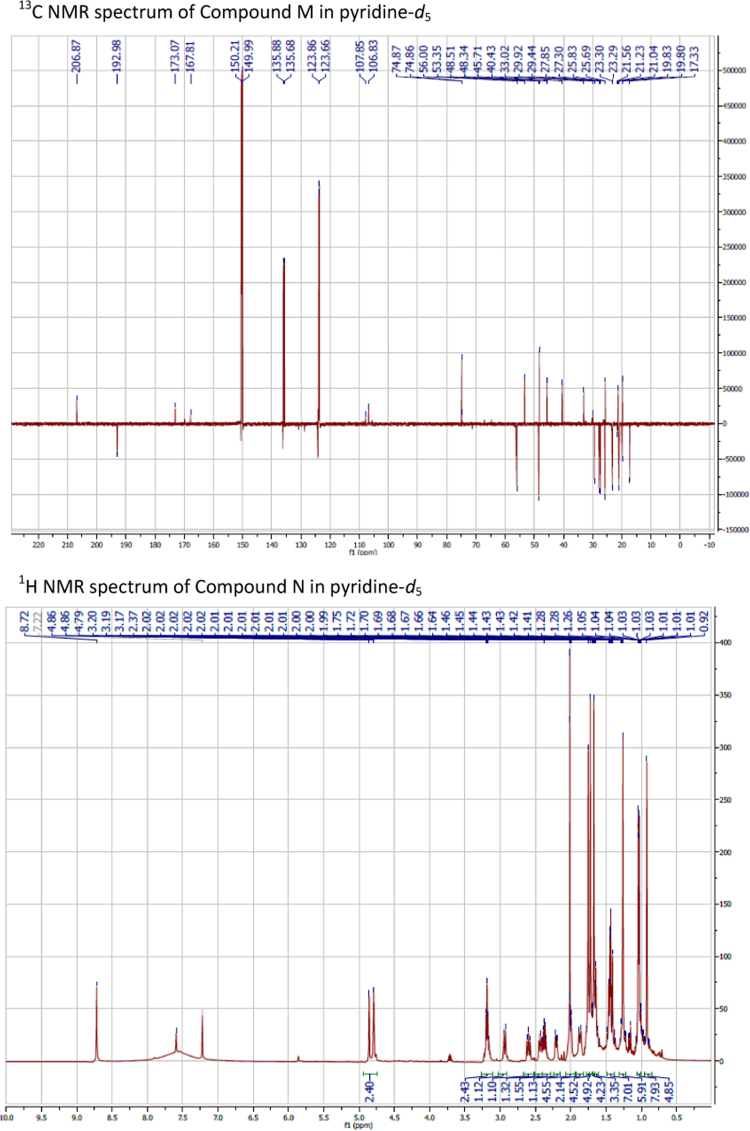

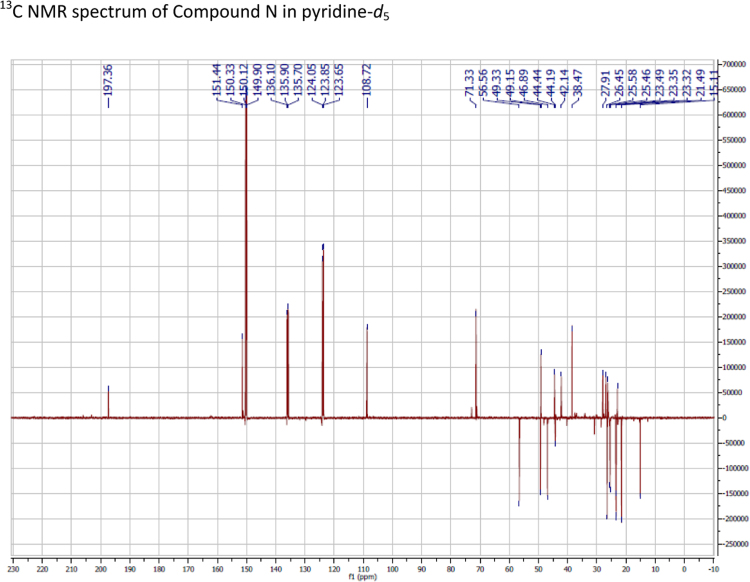
NMR spectra of compounds.

Plant compounds were extracted from the Australian native plants *P. glabrum* Burret (Myrtaceae), *R. psidioides* (G.Don) Benth. (Myrtaceae), *G. parviflora* Lindl. (Rutaceae) and *M. montanum* R.Br. (Scrophulariaceae). The isolation and structural elucidation of these compounds have been described previously [1], [2], [3], [4]. Compound names, structure and references to extraction procedures and analysis of compounds are described in [5]. NMR spectra of compounds are presented in Fig. 1.

### 2.2 In situ immunofluorescence keratin assays

Immortalised human keratinocytes (HaCaTs) were cultured in DMEM with 10% Fetal Bovine Serum (FBS) and penicillin–streptomycin (100 IU/ml penicillin, 100 µg/ml streptomycin) at 37 °C, 5% CO_2_. At confluence HaCaTs were trypsinised and seeded into 96 well plates at 5×10^5^ cells/ml (100 µl per well, 50,000 cells), and cultured for 24 h. Media was removed by aspiration and replaced with serum-free DMEM containing penicillin–streptomycin for 6 h prior to exposure to 20 µM plant derived compounds in serum-free DMEM with penicillin–streptomycin and then incubated at 37 °C, 5% CO_2_ for 60 h. Final DMSO content of media was 0.5% v/v. Test compounds isolated from native Australian plant species were dissolved in neat DMSO prior to addition to media. Negative control was serum-free DMEM with penicillin–streptomycin, and DMSO (0.5% v/v). Blanks were wells containing the same media but no cells. All samples, controls and blanks were performed in replicates of *n*=8.

After 60 h, media was removed via aspiration and washed 3× in Dulbecco׳s phosphate buffered saline (DPBS). Cells were fixed and permeabilized in ice cold methanol for 15 min at −20 °C then washed 3× in PBS. All following incubations were conducted at room temperature. Cells were treated with 0.5% Tween-20 in DPBS (DPBS-tween) for 10 min before blocking with 3% goat serum in PBS-tween for 30 min. Blocking solution was replaced with one of the following primary antibodies (Keratin 1=mouse monoclonal anti-human Keratin 1 (Thermo Fisher Scientific) at 5 µg/ml; Keratin 5=mouse monoclonal anti-human Keratin 5 (Santa Cruz Biotechnology) at 1 µg/ml; Keratin 10=mouse ascites anti-human Keratin 10 (NeoMarkers) at 1 µg/ml; Keratin 14=mouse ascites anti-human Keratin 14 (NeoMarkers) at 1 µg/ml) in blocking solution for 2 h then washed with 3× DPBS. Cells were then incubated with secondary antibody (Alexa Fluor 488 goat anti-mouse IgG (Life Technologies)) at 10 µg/ml in DPBS for 1 h in the dark. Cells were washed with 3× DPBS with a final 100 µl of DPBS added to each well before analysis. Plates/cells were photographed utilising cellSens Dimension software (Olympus) with an Olympus DP80 camera utilising a 1.45MP, 14 bit, monochrome CCD sensor on an Olympus IX83 fluorescence microscope running CoolLED pE light source with 490 nm LED, and a *Märzhäuser* Wetzlar Tango Desktop motorised stage controller.

**Fig. 2:**
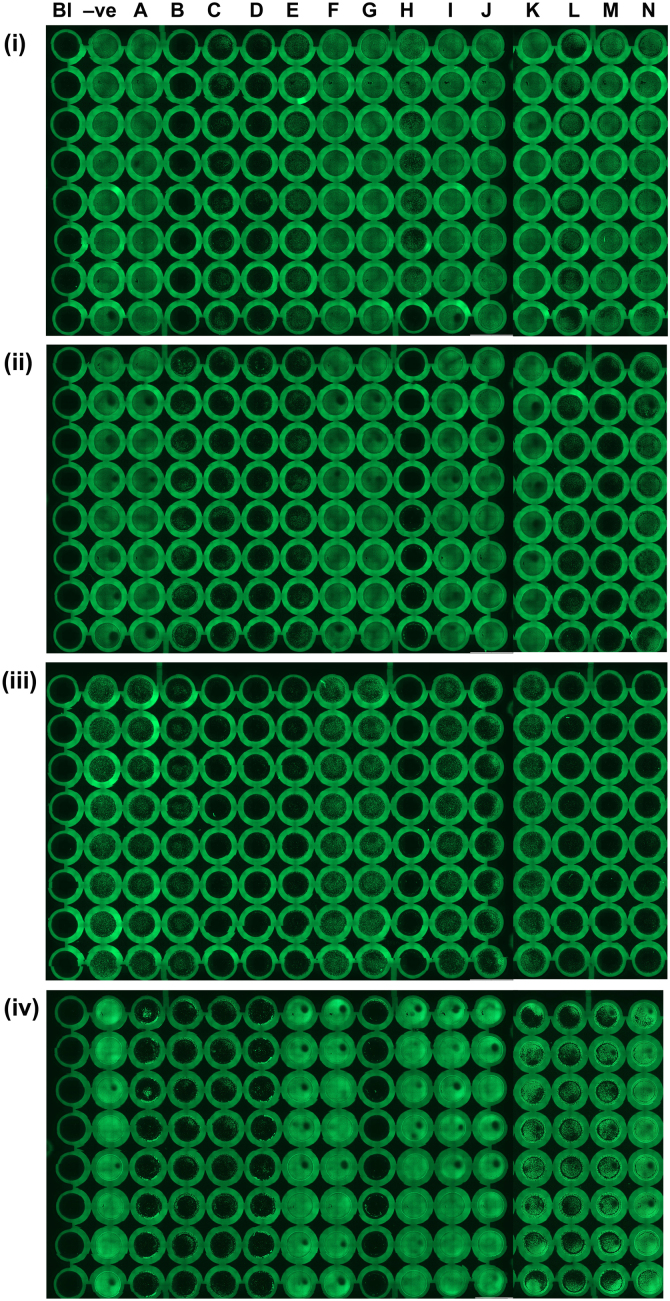
Effect of native Australian plant compounds on the expression of keratin 1, 5, 10 and 14 in cultured human keratinocytes (HaCaTs). Stitched images of keratin expression: (i) Keratin 1; (ii) Keratin 5; (iii) Keratin 10; and (iv) Keratin 14. Column labels are Bl=blank, −ve=negative control, compounds are designated with their respective letters. (A) 1,3,7,9-tetrahydroxy-2,8-dimethyl-4-(2-methylpropionyl)−6-(2-methylbutanoyl)dibenzofuran; (B) rhodomyrtoxin C; (C) 1,3,7,9-tetrahydroxy-4,6-dimethyl-2-(2-methylpropionyl)−8-(2-methylbutanoyl)dibenzofuran; (D) biflorin; (E) (−)-(5R,7R,9R)-Perillup ketol; (F) flindersine; (G) geiparvarine; (H) parvifloranine A; (J) (N-acetoxymethyl) flindersine; (K) rhodomyrtal A; (L) rhodomyrtal B; (M) eucalyptin A; and (N) rhodomyrtal D.

**Fig. 3:**
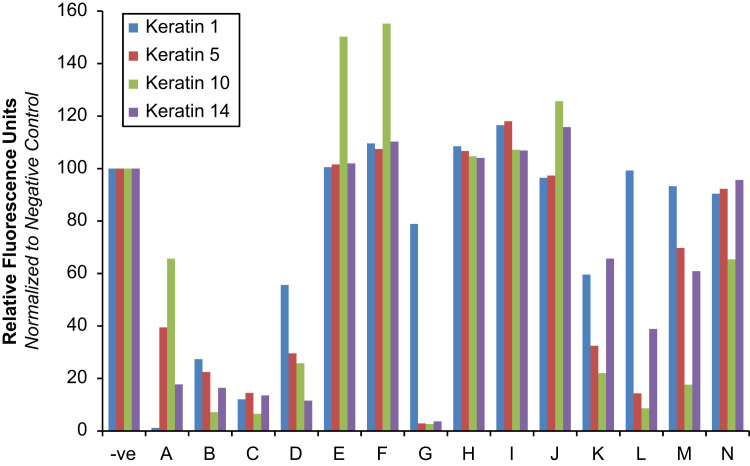
Effect of native Australian plant compounds on the expression of keratin 1, 5, 10 and 14 in cultured human keratinocytes (HaCaTs). Compounds are designated with their respective letters: (A) 1,3,7,9-tetrahydroxy-2,8-dimethyl-4-(2-methylpropionyl)−6-(2-methylbutanoyl)dibenzofuran; (B) rhodomyrtoxin C; (C) 1,3,7,9-tetrahydroxy-4,6-dimethyl-2-(2-methylpropionyl)−8-(2-methylbutanoyl)dibenzofuran; (D) biflorin; (E) (−)-(5R,7R,9R)-Perillup ketol; (F) flindersine; (G) geiparvarine; (H) parvifloranine A; (J) (N-acetoxymethyl) flindersine; (K) rhodomyrtal A; (L) rhodomyrtal B; (M) eucalyptin A; (N) rhodomyrtal D. Results are normalised to negative control (−ve). *P* value<0.05 for all results except Keratin 1=compounds E, J, L; Keratin 5=compounds E, F, H, J, N; Keratin 10=compounds H, I; Keratin 14=compounds E, F, H, I, J, N.

Photographic images of the plate and cells at 40× magnification were automatically stitched together (Fig. 2). Each well had its grey scale intensity value calculated using the cellSens Dimension software with values then normalised to negative control. Keratin levels were calculated in response to treatment with the plant compounds and expressed normalised to negative control (Fig. 3).

### 2.3 Statistical analysis

Statistical differences were determined using the Student׳s *t*-test with two tailed, two-sample unequal variance. A *P* value of less than 0.05 was considered significant.
